# The Impact of Polycyclic Aromatic Hydrocarbons on the Structure and Crystallization Behavior of Nanocomposites Based on Paraffin and Polyethylene

**DOI:** 10.3390/ijms262311509

**Published:** 2025-11-27

**Authors:** Sergey V. Larin, Sofia D. Melnikova, Andrey A. Gurtovenko, Sergey V. Lyulin

**Affiliations:** 1 Branch of the Petersburg Nuclear Physics Institute Named by B.P. Konstantinov of the National Research Center “Kurchatov Institute”—Institute of Macromolecular Compounds, Bolshoi Prospect V.O. 31, St. Petersburg 199004, Russia; 2 Institute of Chemistry, St. Petersburg State University, 7/9 Universitetskaya Nab., St. Petersburg 199034, Russia

**Keywords:** polyethylene, paraffin, polycyclic aromatic hydrocarbons, nanocomposite, computer simulation, molecular dynamics, crystallization

## Abstract

The manufacture of nanocomposite materials based on polyolefins and paraffins is a promising approach for developing materials with enhanced performance characteristics for a wide range of applications. However, a more profound understanding is required to identify how the material properties depend on the type of filler and its concentration, as well as on the structure of the composite matrix. In this study, the effect of adding several types of polycyclic aromatic hydrocarbons (PAH) with different molecular sizes—coronene, ovalene, and hexabenzocoronene (HBC) at concentrations ranging from 10 to 40 wt%—to composites based on polyethylene (PE) or paraffin was studied using all-atomistic computer simulation. Our study revealed how the size of the PAH molecules influences their aggregate behavior, phase transitions, and the structure of the composite matrix. It was shown that coronene exhibits weak aggregation in paraffin and prevents its crystallization, which results in a pronounced decrease in the crystallization temperature of both PE and paraffin in the composites. Ovalene and HBC demonstrate a stronger tendency to aggregate, forming substantially larger aggregates than coronene. Both these PAHs increase the crystallization temperature of PE or paraffin in the composites. At the same time, up to a certain concentration, ovalene can integrate into the paraffin crystal structure at low temperature, promoting its crystallization. In contrast, HBC forms extended columnar aggregates at both high and low temperatures, creating steric hindrances to the formation of the crystalline matrix structure and ultimately reducing the degree of crystallinity of PE or paraffin in the composites. Thus, by adding aromatic nanofillers, it becomes possible to control the microstructure and phase behavior of polyolefin-based composites.

## 1. Introduction

Polyethylene-based composites filled with carbon fillers are widely used today in a range of industries where there is a requirement for enhanced performance characteristics compared to unfilled polyethylene (PE). Such materials can be used in packaging products [1,2], to produce storage devices for electrical [3,4,5,6,7] and thermal energy [8,9,10,11], for water purification [12,13,14], in the biomedical [15] and aerospace industries [16,17].

The specific physical characteristics of composites are mostly determined by the type and amount of filler being introduced. Out of the whole cornucopia of available fillers in recent decades, carbon nanofillers of varying types have been deemed the most promising [18,19,20,21], including carbon black, fullerenes, nanotubes, nanofibers, graphene, as well as structurally similar natural molecules with a high carbon content, such as asphaltenes [22,23,24]. Moreover, asphaltenes represent an extremely attractive type of filler for polyethylene, given that they are a cheap by-product of oil refining, and so do not significantly increase the cost of composite materials, while at the same time improving their mechanical and thermophysical properties.

The addition of carbon nanoparticles into polymers makes it possible to modify in a controlled manner the materials’ mechanical characteristics (their modulus of elasticity, wear resistance, etc.), as well as to obtain electrically conductive materials and composites with increased heat resistance and thermal conductivity [19,20,25,26,27,28,29,30]. At the same time, however, there is frequently a reduction in the strength of the material and an increase in its tendency to crack [26,27,28], thereby narrowing the scope of their application, especially in the manufacture of composite membranes and films.

The introduction of plasticizers into composites to increase their plasticity can be considered as a promising way to solve this problem. In the case of PE and other polyolefins, such as paraffins and waxes (i.e., polyolefins with a lower molecular weight), they can be used as potential plasticizers [31,32,33,34]. The similar chemical structure of the polymer and plasticizer in this case ensures good compatibility of the material’s components, preventing a decrease in its performance due to microphase segregation of the material. It is also worth noting that blends of PE with paraffins or the same blends with the addition of carbon particles can be used as phase-transition materials for the production of thermal energy storage devices [8,10,11,35]. At the same time, the addition of polyethylene ensures the structural stability of the material, while the introduction of carbon nanoparticles is necessary to increase its thermal conductivity [36,37,38].

Thus, industrial composites based on PE can be considered as a complex system consisting of a polymer matrix, a plasticizer, and a filler. Each of these components contributes to the final properties of the material, exerting mutual influence that must be taken into account when developing new materials for industrial applications. The impact of the individual components on the microstructure and thermal behavior of the composites is particularly important from the standpoint of practical use of polyethylene-based materials. For example, the crystallization of PE can significantly affect the mechanical properties of the materials derived from it; changes in the phase-transition temperatures caused by the addition of plasticizers or nanoparticles influence the potential application range of the composites; and modifications in the aggregation behavior of nanoparticles play a decisive role in determining their functional characteristics, including changes in electrical and thermal conductivity.

For PE-based composites using carbon nanotubes as a filler, R. Haggenmüller et al. demonstrated an increase in the crystallization rate of the PE and a change in the growth pattern of crystallites even at an extremely low filler content [39]. It was shown that in composites with 1 wt% of carbon nanotubes, the half-time of crystallization was approximately 7% of this for neat HDPE. Using the example of composites based on ultrahigh molecular weight PE filled with carbon nanotubes, G. Xu et al. showed the effect of nanoparticles on the nucleation of polymer crystallization, although the crystallization temperature and the rate of crystallite growth in this case turned out to be close to the parameters for an unfilled polymer [40]. In composites containing graphene as a filler, A. Abuibaid et al. observed nucleation of crystals on the surface of nanoparticles and a higher crystallization rate compared to unfilled PE [41]. However, E. Tarani et al. observed that an increase in graphene concentration in composites leads to a decrease in the mobility of the polymer matrix chains and, as a result, complicates the crystallization of the PE, especially at the final stages of the process [42,43,44].

The dependence of polymer matrix crystallization on filler concentration is a critically important factor governing material properties. The concentration range of filler from 10 wt% to 40 wt% considered in our study is typical for composites filled with various mineral composites [45]. For nanocomposites, the large surface area of interactions between the filler and the matrix usually allows to decreasing filler content to 3–5 wt%. However, in the case of strong aggregation of nanoparticles in material, the interaction surface area decreases significantly, and the increased nanofiller concentrations should be used to achieve notable improvement of composite properties.

For polyethylene-based composites, an increase in matrix crystallinity with filler concentration is often observed. Such dependencies have been reported, for example, for composites containing small-diameter graphene nanoplatelets (GNP) at concentrations up to 5 wt% [42,44] and chalk (up to 25 wt%) [46]. However, an increase in GNP size alters the nature of this dependence, rendering it non-monotonic. In this case, the nanoparticles exhibit an additional nucleation effect only at low concentrations (up to 2.5 wt%) [44]. A further increase in concentration leads to a decrease in crystallinity, which is attributed to decreased PE chain mobility due to interactions with graphene particles and, consequently, restricted crystallite growth. The same non-monotonic dependence was also observed for marble-filled composites, where a decrease in PE crystallinity was found at filler concentrations above 5 wt% due to the bigger size of filler particles in comparison with chalk-filled composites [46]. Also, N. Vidakis et al. have shown that the maximum effect of carbon black filler on the thermal, mechanical, and rheological properties of HDPE-based composite is observed at a concentration range up to 20 wt% of filler [47]. Furthermore, for composites containing silicon oxide (silica) nanoparticles, a monotonic decrease in polyethylene crystallinity was observed. This decrease is caused both by enhanced interactions between the composite components at the interface and by spatial constraints associated with the formation of a nanoparticle network [48]. Thus, the dependence of polyethylene crystallinity on filler concentration in a composite is determined by a combination of factors related to the interactions between the material components and the resulting spatial restrictions on polymer chain mobility.

For paraffins a significant effect of the addition of nanoparticles on phase transitions and thermophysical properties of materials has also been shown. S. Srinivasan et al. found that the addition of graphite particles (3.5 wt%) leads to a 450% enhancement of the thermal conductivity of n-eicosane and reduced interfacial thermal resistance [49]. A study of paraffin-based composites filled with asphaltenes reported by V. Makarova et al. shows that this type of filler leads to a decrease in the paraffin melting point and degree of crystallinity as well as a slight increase in the thermal conductivity of the composite [50]. Interestingly, it was found that modified asphaltenes could provide an increase in the thermal conductivity of the composite with maximum effect at a filler concentration close to 20 wt%. In the research carried out by Y. Zhao the sufficient effect of filler particle size on the composite properties was found, i.e., an increase in thermal conductivity of the composite up to 1695% upon addition of large particles of expanded graphite [51]. It is worth noting that the addition of paraffins to polyethylene can also lead to a change in its phase behavior [33,34,52,53].

It follows from the above that predicting the properties of composite materials of complex formulation requires a simultaneous understanding and consideration of the influence of nanoparticles, whether on the structure of the paraffin or of the polyethylene, as well as on the phase transitions of both components of the composite matrix. It is also important to take account of the effects related to the size of the nanoparticles introduced as a nanofiller into the composite. This is due to the fact that promising natural low-cost fillers (such as the aforementioned asphaltenes) are a complex mixture containing molecules of different sizes and shapes, which may exhibit different aggregation behavior and have varying effects on the microstructure of the polymer-based blends. Accordingly, the presence or absence of effects associated with the size of filler nanoparticles will help to understand better the mechanisms altering the properties of nanocomposites, and to predict and constructively modify these properties.

One of the most cutting-edge approaches to analyzing and predicting the properties of polymer materials at the molecular level is computer simulation using the molecular dynamics method. The current state of the art in terms of computational methods, software, and hardware makes it possible to simulate complex polymer systems on a microsecond time scale in a relatively short time [54,55,56], which is simultaneously essential to obtaining correct equilibrium configurations for the systems being studied [54,56], as well as enabling us to study even the slowest processes in polymer systems, including crystallization [57,58,59,60]. A fairly large number of papers have been devoted to the study of the crystallization of paraffins and polyethylene using computer simulation. H. Zerze et al. investigated the formation and growth of crystal nuclei in n-alkanes using molecular dynamics, obtaining a good correlation between the simulation results and experimental data in terms of the density and parameters of the crystal lattice of the nuclei being formed [61]. The crystallization of n-eicosane under conditions of shear deformation and stretching was studied using non-equilibrium molecular dynamics in the work of D. Nicholson et al., making it possible to obtain data on the dependences of crystallization kinetics on the conditions of paraffin deformation [62]. In a series of studies by T. Yamamoto, a comprehensive investigation of PE crystallization was undertaken using coarse-grained united atoms models, including the formation of crystallization nuclei in polyethylene, both in separate chains in vacuo and in a melt [63]; the growth of the PE crystalline phase from the melt, including on the surface of the crystalline phase [63,64]; and the crystallization of PE during the fiber formation process and under shear deformation [65]. The mechanisms of crystallization in melts of n-alkanes and short chains of PE were also studied using mesoscopic models in the research of M. Anwar et al. [66,67]. It should be noted that these studies examined the crystallization of PE and alkanes mainly under isothermal conditions in a supercooled state (i.e., at a temperature below the crystallization temperature), which does not enable us to fully assess the dynamics of phase transitions. The non-isothermal crystallization of PE was considered in the work of J. Ramos et al. [68], where a good correlation was obtained between the simulation results and experimental data, including the dependence of the crystallization process on the cooling rate of the melts, although coarse-grained united atom models of PE were used in this work. It is worth noting that in the results of molecular dynamics simulations of blends of PE with paraffins published to date, a conclusion is drawn regarding the partial co-crystallization of paraffin with PE. However, these conclusions are based on indirect evidence from temperature transitions in the small-scale systems studied, which were obtained at extremely high cooling rates [53,69].

It should be pointed out that only few studies using computer simulation methods exist which deal with crystallization of a matrix in composites based on PE filled with carbon nanoparticles. Moreover, it is frequently the interaction of single PE chains with nanoparticles that is examined [70,71,72], or the systems are of a very small size, and with short simulation times insufficient for studying the crystallization of the polymer matrix in the composite. In particular, in the work of L. Liu, systems containing up to 140 PE chains with a degree of polymerization of *n* = 20 and a single filler particle with a size of approximately 3 × 3 nm were considered, and the simulation times were up to 2 ns [73]. H. Yazdani et al. conducted the simulation of composites based on long PE chains (with a length of 1000 united atoms), but the total simulation time of the studied systems was close to 100 ps only [74]. Also, in the work of Z. Yang et al., systems based on short PE chains with a degree of polymerization of *n* = 30 with a total system size of 3 × 3 × 3 nm^3^ were considered, and the simulation times were about 2 ns [75].

In a series of recent studies carried out in our group, a detailed and intensive investigation of the crystallization processes in n-eicosane and its blends with asphaltenes of various compositions was carried out on a microsecond timescale. The studies made it possible to determine the influence of the choice of the paraffin model [76] and the cooling rate of the systems [77] on the phase transitions occurring, in order to study the influence of asphaltenes on the crystallization of paraffin and the composite structure as a whole [78,79].

The present work is a logical continuation of the previous research cycle. It focuses on a comparative study of the effects of carbon nanofillers of different molecular sizes on the structure and phase transitions of polyethylene- and paraffin-based composites at various filler concentrations. In addition, the aggregation behavior of the filler molecules is analyzed. These investigations were carried out using molecular dynamics simulations. Several types of polycyclic aromatic hydrocarbons (PAHs)—namely coronene, ovalene, and hexabenzocoronene (HBC)—were examined as fillers in this study. The choice of PAHs as fillers is due to the fact that they are ideal model nanoparticles, possessing structural similarities to typical carbon nanoparticles (graphene and asphaltenes), but at the same time have a strictly defined chemical structure. Moreover, the PAH molecules selected differ in size and shape, which makes it possible to assess the influence of these factors on the properties of the composites being studied.

We should note here that the PAHs could be quite different in size, aspect ratio, or surface chemistry with graphene nanoplatelets. Since the precise chemical structure of graphene nanoparticles or, for example, asphaltene molecules cannot be determined experimentally, it often constitutes a variable factor in research. Thus, the choice and validation of model systems invariably present a significant challenge when investigating the factors governing composite structure. From that point of view, the well-defined structure of PAHs made them ideal model substances to establish the most fundamental principles and factors determining the properties of composite materials. It is worth noting that the approach of using PAH as model molecules in the creation and study of the properties of polymer composites is frequently used not only in computer modeling, but also in experiment-based research [80,81].

## 2. Model and Simulation Technique

**Figure 1:**
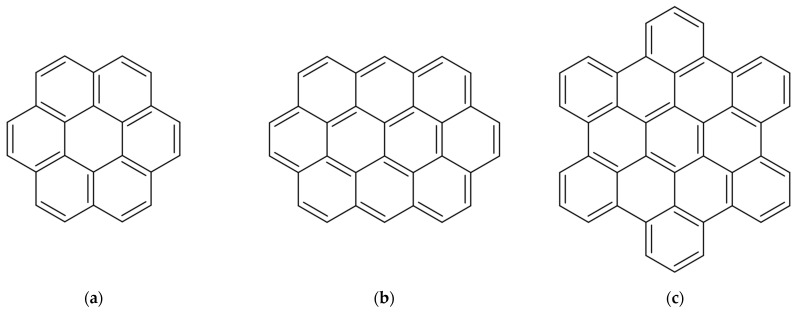
Chemical structure of polycyclic aromatic hydrocarbons (**a**) coronene, (**b**) ovalene, and (**c**) hexabenzocoronene.

The study investigated the structure and phase transitions during cooling for nanocomposites based on either saturated hydrocarbon n-eicosane (C_20_H_42_) or linear polyethylene with a degree of polymerization *n* = 250 (C_500_H_1002_), filled with polycyclic aromatic hydrocarbons with varying aromatic core sizes: coronene (C_24_H_12_), ovalene (C_32_H_14_), and hexabenzocoronene (HBC, C_42_H_18_), the structural formulae for which are shown in Figure 1. These PAHs can be viewed as model equivalents of modified asphaltenes without lateral aliphatic groups, the properties of whose blends with paraffin have been examined in our previous studies [78,79]. The polyethylene model is structurally analogous to HDPE but features a reduced molecular weight compared to the industrial-grade polymer, consistent with the standard practice in computer simulation of polymers.

In order to examine in detail the structure and properties of the composite systems being studied, full-atomistic molecular dynamics computer simulation was performed using the GROMACS 2021.6 software package [82,83,84]. The simulation was carried out using the CHARMM36 force field [85,86]. Analysis previously conducted by us as to the applicability of various models and force fields for studying the properties of paraffins using molecular dynamics methods showed that it is this force field that gives optimal correlation between the results of computer simulation and experimental data in terms of the structural and thermophysical properties of paraffins [76]. Additionally, the CHARMM36 force field has previously been used successfully for composites based on paraffin and asphaltenes [78,79]. In the CHARMM36 force field the forces acting on each atom are derived from bonded and non-bonded interactions between atoms. Non-bonded interactions include van der Waals (excluded volume) and Coulomb (electrostatic) interactions between atoms not connected by valence bonds. Bonded interactions take into account forces related to deformation on valence bonds and valence angles between atoms as well as internal rotation around valence bonds (deformation of dihedral angles).

Each system studied consisted of 500 n-eicosane molecules or 20 PE chains, as well as one of the three types of PAH being studied. In the composite systems examined, a certain number of PAH molecules (N_PAH_ = 44, 99, or 214) were added to the cubic simulation cell, similar to the earlier study for systems containing n-eicosane and asphaltene molecules [78,79]. This amount of PAH corresponded to the range of mass concentrations of the filler from ~10% to ~40%, and it was in this concentration range that the most significant influence of asphaltenes on the properties of paraffin-based blends had been previously observed. The composition, mass fraction of the fillers and characteristic sizes of the systems studied are shown in Table 1.

The simulation procedure was similar to that previously used for paraffin-based systems [76,78,79]. At the first stage, matrix molecules (n-eicosane or PE) and the PAH were added to the cubic simulation cell. For each of the systems volumetric compression in the NpT ensemble was performed for 5 ns at a pressure of 50 bar and a temperature of 450 K. This simulation temperature was chosen both to speed up the equilibration of the systems studied and to investigate their structure in the molten state. The temperature of 450 K is sufficiently higher than the melting point of PE and n-eicosane. Although the onset decomposition temperature for n-eicosane is slightly below 450 K, the maximal rate of decomposition is observed at higher temperatures, close to 490 K [87], and experimental data on the n-eicosane density and thermal properties are reported up to temperature 450 K [88].

**Figure 2:**
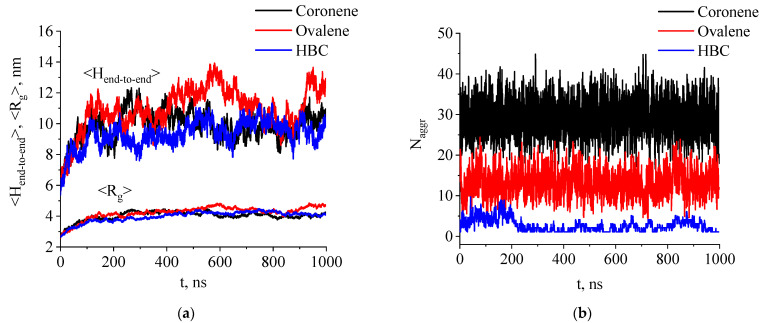
(**a**) Average end-to-end distance H_end-to-end_ and gyration radius R_g_ for PE chains in composite systems with different filler types (coronene, ovalene, and HBC) depending on simulation time where N_PAH_ = 99; (**b**) average number of PAH aggregates N_aggr_ in systems based on PE with different filler types depending on modeling run time where N_PAH_ = 99. Data shown for simulations run at a temperature of 450 K.

The pressure was then reduced to 1 bar, and the systems were simulated for 1 microsecond, corresponding to the stage of preliminary modeling (equilibration). Analysis of changes in the size of the polyethylene chains and the size of the PAH aggregates in the composites showed that equilibrium was achieved during the first 200 ns of modeling. After this time, only fluctuations in the gyration radius and the end-to-end distance of the PE chains were observed, as well as the size of the PAH aggregates in the composites close to the average value (Figure 2).

As the crystallization temperature and maximum crystallinity of the composite matrix could depend sufficiently on the cooling rate, its choice was made based on our previous extensive work devoted to the investigation of the cooling rate effect on the phase transition in paraffin [77]. The cooling rate of 10^8^ K/s (6·10^9^ K/min) was found to be most appropriate to study the crystallization process in paraffin-based systems and was used for PE- and paraffin-based composites in the current research. It should be noted also that this cooling rate is almost the slowest achievable in simulation nowadays. Further decrease in cooling rate requires tremendous computational resources and is not practical.

The simulation procedure we used in our research allows us to investigate the crystallization process in the composites considered using a technique that mimics a non-isothermal crystallization regime. All systems were cooled from a temperature well above the melting point (450 K) to a temperature well below their crystallization temperature (250 K). The simulations were performed at a cooling rate sufficiently low to observe the crystallization process [77]. Thus, the structure of the systems at 250 K corresponds to the completed crystallization process, and comparative analysis of the two types of composites at this specific temperature allows us to identify the structural features of the materials, which depend on both the type of composite matrix and the type of filler.

It should also be noted that below the crystallization temperature, the mobility of the components in the studied systems becomes rather low, and neither prolonged simulation at this temperature nor further cooling (including below the glass transition temperature) would result in significant structural changes to the composites compared to the results of this work.

In order to evaluate the aggregation behavior of the PAH in the composites, the average number of aggregates N_aggr_ formed by the PAH molecules, and their average sizes N_av_ (the average number of PAH molecules included in one aggregate), were calculated. The size and number of the PAH aggregates were determined using cluster analysis. In order to assign the PAH molecules to a specific aggregate, a geometric criterion was used [89,90], according to which two PAH molecules were considered to belong to the same aggregate if the minimum distance between their atoms did not exceed a given critical distance of 0.45 nm [78].

In order to determine the effect of the addition of the PAH to the composites on the crystallization of n-eicosane and PE for all the composites studied, the degree of crystallinity of the composite matrix was calculated using the approach proposed by T. Yamamoto et al. [65]. To assess the degree of crystallinity of the systems studied, the simulation cell is divided into cubic regions with a volume of (2σ)^3^, where σ = 0.38 nm is the Van der Waals diameter of carbon atoms. For each of these regions a local order orientation parameter is calculated $P_{2}\left(\vec{r}\right)$:

(1) $$P_{2}\left(\vec{r}\right)=\left\langle \frac{3}{2}{cos}^{2}\theta -\frac{1}{2}\right\rangle ,$$

where *θ* is the angle between vectors connecting the centers of the adjacent C-C bonds within a single cubic region at position $\vec{r}$. An average value was calculated for all bonds within the same region, and a region was deemed to be crystalline where the value of the order orientation parameter $P_{2}\left(\vec{r}\right)$ for the C-C bonds was greater than 0.7 [65]. The value of the system’s crystallinity χ was determined as being the ratio of the number of crystalline cubic regions to the overall total number of regions containing C-C bonds within the system.

## 3. Results and Discussion

### 3.1. Aggregation Behavior of PAH in PE and Paraffin

**Figure 3:**
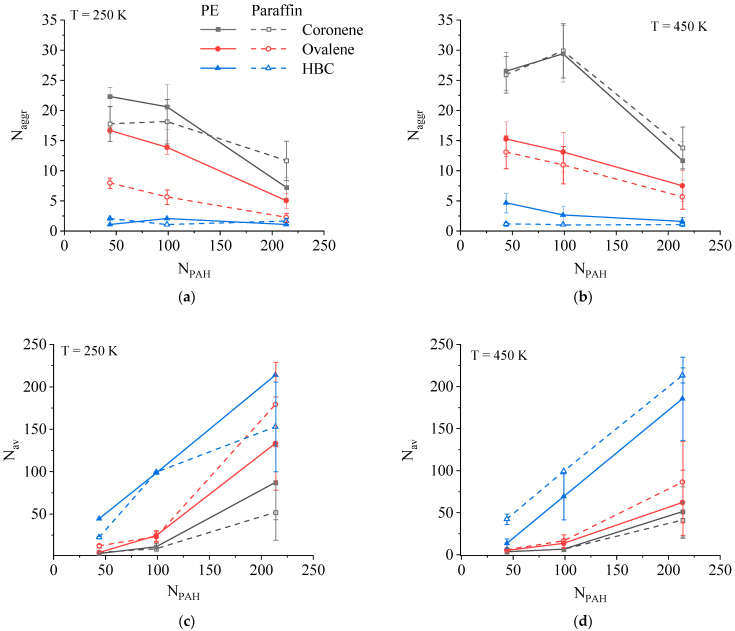
Number of aggregates N_aggr_ (**a**,**b**) and average aggregate size N_av_ (**c**,**d**) of the PAH in the composites studied where T = 250 K (**a**,**c**) and 450 K (**b**,**d**).

Analysis of the aggregation behavior based on the concentration dependencies of the average number of PAH clusters N_aggr_ in the systems studied and the average cluster size N_av_ in PE and paraffin-based composites generally showed similar results for both types of composites (Figure 3). At the same time, the PAH aggregation turned out to depend primarily on the size of the PAH molecules, but not on the degree of polymerization of the matrix.

For all systems in the melt state, the size of the PAH clusters increases with both an increase in the size of the PAH molecules and an increase in the concentration of the PAH. At a low temperature of 250 K, these dependences also persist, with the single exception of paraffin-based composites with ovalene and HBC at N_PAH_ = 214, in which the cluster size in the systems with ovalene is larger than for the composite with HBC. As will be shown below, this behavior is related to the features of the formation of the structure of paraffin + ovalene composites during crystallization.

**Figure 4:**
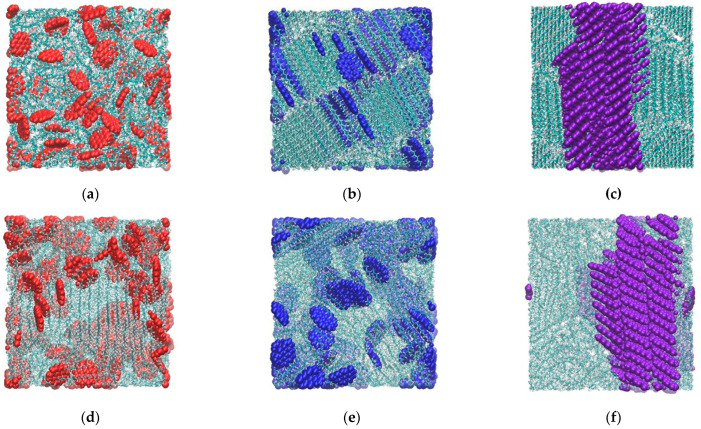
Snapshot configurations of composites based on paraffin (**a**–**c**) and PE (**d**–**f**) with coronene (**a**,**d**), ovalene (**b**,**e**), and hexabenzocorene (**c**,**f**) at temperature T = 250 K. Quantity of PAH molecules in composites N_PAH_ = 99.

Hexabenzocoronene (HBC) molecules form large aggregates both in the melt at 450 K and at 250 K. An increase in the concentration of HBC leads to an increase in the size of the aggregate, which includes almost all the filler molecules available in the system. During cooling, the average size of the aggregates remained virtually unchanged in both paraffin and PE. In the case of high filler concentrations, the aggregates had a columnar structure consisting of several extended stacks interacting with each other (Figure 4c,f). A similar pattern was observed earlier for systems based on paraffin and modified asphaltenes without lateral aliphatic groups [78,79].

For the ovalene molecules in systems at 450 K, the average size of the aggregates was relatively small. Meanwhile, in composites where the number of ovalene molecules was 99 or fewer, embedding of the ovalene molecules into the crystal structure of the paraffin was detected as the temperature decreased. There was moreover a tendency for the ovalene molecules to be arranged in parallel to the ordered chains of n-eicosane, most frequently as two molecules in a line along the largest axis of the ovalene (Figure 4b). A significant role in the formation of such a structure may be played by the fact that the distance between the ends of the n-eicosane chain in the ordered state is approximately two times the size of the ovalene along the largest axis. With an increase in the ovalene content in the paraffin-based composite to N_PAH_ = 214, the formation of a single columnar structure aggregate was also observed, as was the case for HBC, moreover the aggregate was already formed at a temperature of 450 K, and a subsequent decrease in temperature did not result in its destruction and the incorporation of the ovalene molecules into the crystal structure of the paraffin. In the PE-based composites, such incorporation of ovalene into the crystal structure of the matrix was not observed. This may be due to the fact that in the PE-based systems, due to its longer chains, the degree of crystallinity of the systems is lower, and the ordered regions are more chaotically arranged relative to each other than in the case of paraffin, where a structure with a layered arrangement of crystalline domains is formed.

When coronene is introduced, no formation of large aggregates in either type of composite is detected, either at 450 K or at 250 K. In the case of this PAH, an almost uniform distribution of molecules is generally observed over the entire extent of the composite (Figure 4a,d). Only at a high concentration of coronene in PE is the formation of small aggregates observed at low temperatures, whereas at 450 K, the aggregation behavior of coronene is almost identical for PE and for paraffin. The formation of coronene aggregates in PE at a temperature of 250 K is due to the fact that coronene molecules are located mainly in the amorphous regions of the partially crystalline PE. This leads to a local increase in the density of this PAH in those areas and, as a result, an observed increase in the number of aggregates. Separately, we note that for composites with coronene as a filler, there is a pronounced non-monotonic dependence of the number of PAH aggregates on the filler content in the system. This generally indicates a low tendency of the coronene molecules to aggregate in blends with paraffin or polyethylene, which leads to the formation of a large number of small aggregates at low filler concentrations in these systems.

The aggregation behavior of the filled molecules should be closely related to the compatibility of components in the composites, especially in the melt state. To establish a relation between the aggregation of PAHs in the PE- or paraffin-based composites, the Hildebrand solubility parameters δ were estimated at simulation temperature T = 450 K. Solubility parameters were obtained using the results of simulations of pure systems containing only PE, paraffin, or the certain PAH using the equation

(2) $$\delta =\sqrt{\frac{E_{coh}}{V_{M}}},$$

where *E_coh_* is the intermolecular interaction energy, *E_coh_/V_M_*—cohesive energy density, *V_M_* = *M*/ρ—molar volume of the system, ρ and *M* are the density and the molar mass of the system. In turn, intermolecular interaction energy can be calculated as:

(3) $$E_{coh}=\sum_{N}E_{is}-E_{tot,}$$

where *E_is_* is the potential energy of individual molecule, *E_tot_* is the total potential energy of the system calculated in molecular dynamics simulation, and *N* is the number of molecules in the system [91].

It was found that the solubility parameters are similar within each group—paraffin (δ = 12.9 (J/cm^3^)^0.5^) and polyethylene (δ = 13.8 (J/cm^3^)^0.5^) on the one hand, and the three PAHs—coronene (δ = 20.4 (J/cm^3^)^0.5^), ovalene (δ = 20.6 (J/cm^3^)^0.5^), and HBC (δ = 22.2 (J/cm^3^)^0.5^)—on the other. The solubility parameters obtained show that the compatibility of PE and paraffin with PAH_S_ are very similar. The rather large difference between solubility parameters of aliphatic and aromatic components corresponds to quite low thermodynamic compatibility of components and explains the formation of large aggregates of ovalene and HBC in composites at large concentrations. The uniform distribution of coronene in the composites can be related to the smaller size of this molecule in comparison with two other PAHs and its higher mobility in the melt. At the same time, similar compatibility of PAHs with both PE and paraffin leads to very similar aggregation behavior of the filler molecules at the systems corresponding to the melt state (T = 450 K) as it follows from Figure 3b,d.

An additional factor that can affect the aggregation and crystallization behavior of the composites studied is the ratio between the viscosities of the composite components. This factor is taken into account in our simulations naturally due to most detailed atomistic models and an extensively validated force field utilized. According to the results obtained we can state that the main changes in aggregation of PAHs observed in our study are related to the matrix crystallization process but not to the system viscosity directly. First of all, it is confirmed by the similar aggregation of PAHs in PE and paraffin at the melt state. Also, the additional analysis of aggregation behavior of PAHs in paraffin-based composites shows almost the same aggregation of PAHs at T = 450 K (high above composite crystallization point) and T = 350 K (just prior crystallization beginning) where melt viscosity differs significantly (Figure S1 in the Supporting Information).

Overall, based on our findings, we can conclude that the observed differences in aggregation of PAH in the solid state of the composites studied can be due to the differences in crystallization processes in paraffin- and PE-based composites and will be discussed in the following sections.

### 3.2. Crystallinity of the Composite Matrix

**Figure 5:**
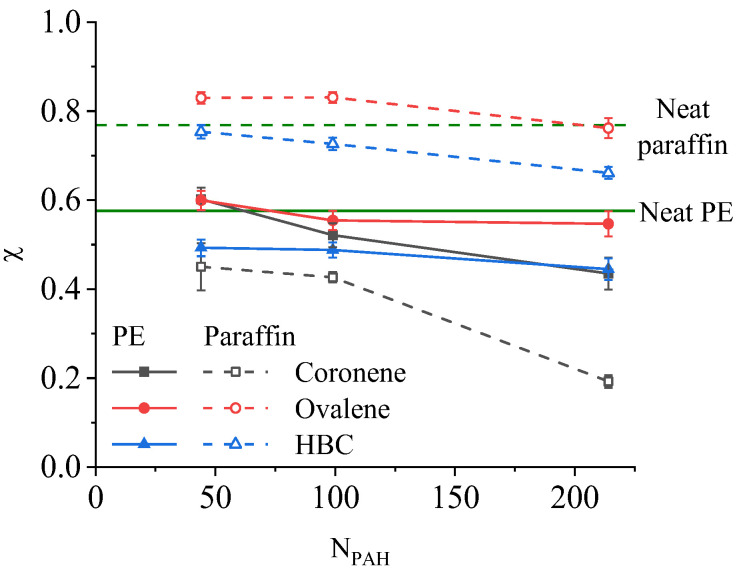
Degrees of crystallinity χ for n-eicosane and PE in composites containing PAH at a temperature of 250 K. The dotted horizontal line indicates the degree of crystallinity for neat, unfilled paraffin, while the solid horizontal line indicates the degree of crystallinity for neat PE.

As mentioned above, in the case of the paraffin-based composites, due to the smaller size of the matrix molecules and their greater mobility in computer simulation, it is possible to achieve almost complete crystallization of the paraffin during cooling, whereas in the case of PE only partial crystallization can be achieved due to the low crystallization rate. Evaluation of the degree of crystallinity of the matrix χ in the composites studied showed a strong dependence on the type and concentration of filler for the paraffin-based systems (Figure 5).

Generally speaking, an increase in the filler concentration leads to a gradual decrease in the degree of crystallinity of the paraffin, although this decrease is due to various factors depending on the size of the PAH molecules. Accordingly, the crystallinity of the paraffin at 250 K in the systems with 44 and 99 HBC molecules was approximately the same as in the unfilled sample, while upon the introduction of 214 HBC molecules into the system, the crystallinity decreased by ~10%, primarily due to an increase in the size of the PAH aggregates that interfere with the orientation of the paraffin molecules. For the systems containing ovalene in quantities up to N_PAH_ = 99, the degree of crystallinity of the paraffin increased by ~10–15% compared with neat, unfilled paraffin, while at N_PAH_ = 214 there was no change. This increase in the degree of crystallinity for these composites is associated with the tendency of ovalene molecules to integrate into the crystal structure of paraffin (Figure 4b), as mentioned earlier, which serves as an additional orienting factor during crystallization. At the same time, the degree of crystallinity of the paraffin upon the addition of coronene, the molecules of which are least likely to form aggregates, and are distributed throughout the volume of the composite, thereby preventing the orientation of the paraffin, decreased significantly, and was close to zero at the maximum filler concentration.

In the case of the polyethylene-based composites, it transpired that the degree of crystallinity of the matrix at 250 K is slightly less dependent on the concentration of the filler. Only for the composites filled with coronene was there a significant decrease in the degree of crystallinity with an increase in its content in the system, whereas for ovalene and HBC, the degree of crystallinity remained almost constant. Moreover, in the case of the PE-based composites containing coronene (at a low concentration of N_PAH_ < 99) or ovalene as a filler, the degree of crystallinity of the polymer remained at a level close to the degree of crystallinity of unfilled PE. In addition, at all concentrations of coronene the decrease in the crystallinity of the PE in the composite was significantly less than in the case of adding coronene to paraffin. For systems with HBC, the degree of crystallinity turned out to be slightly lower at all the concentrations studied than the degree of crystallinity of the unfilled polymer, which, as in the case of paraffin, is associated with the formation of large HBC aggregates that interfere with the ordering and crystallization of the matrix. However, unlike paraffin, a decrease in the degree of crystallinity of PE is observed at all concentrations of HBC, and not only at the maximum. This effect may be due to the fact that in the case of PE it is more difficult for longer polymer chains to reorient and arrange themselves in the presence of large PAH aggregates, in contrast to the more mobile short paraffin molecules.

It should be noted that the results obtained are in good agreement with the experimental data obtained by E. Tarani et al., whose work on PE composites with graphene particles of various diameters showed that large graphene nanoparticles reduce the rate of crystallization of PE by limiting the mobility of polymer chains and their ability to mutually re-order [42,43].

### 3.3. Influence of PAH on the Phase Transition of Composites

**Figure 6:**
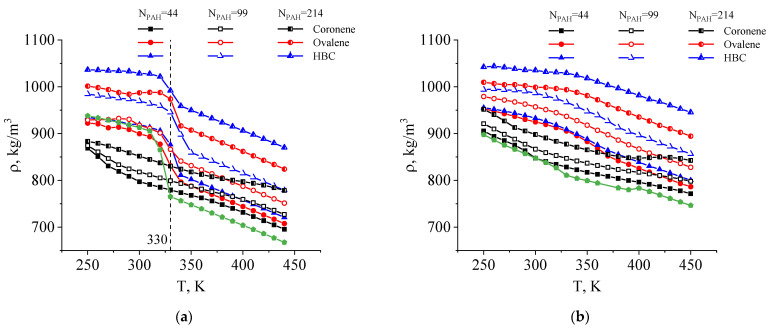
Cooling curves for composites containing PAH based on paraffin (**a**) and PE (**b**). The green symbols indicate the temperature density dependence for neat paraffin and PE.

In order to examine the processes associated with phase transitions in the systems studied, their incremental cooling was modeled, and their density versus temperature dependences were obtained (Figure 6).

For all the systems, an increase in density was observed alongside an increase in filler concentration, with maximum density being typical for systems with HBC, and minimum values for composites using coronene as a filler.

In the cases of the paraffin-based composites containing HBC and ovalene as a filler, density curves characteristic of a crystallization process in the composite matrix were obtained upon cooling: they displayed a sharp jump in density in the region of the phase transition temperature (T ≈ 330 K). An increase in the filler concentration has practically no effect on the temperature of the phase transition for the systems studied. However, the introduction of coronene did not result in any abrupt change in density corresponding to crystallization, which is associated with a significant deterioration in the ability of paraffin to crystallize with the addition of coronene and, as a consequence, it proved impossible to evaluate the characteristics of the phase transition of paraffin in these systems based on density versus temperature dependence.

For the PE-based composites, the dependence of density on temperature during system cooling is more complex. In the case of the PE composites with coronene, just as with the paraffin composites with coronene, a section with a shallow gradient (corresponding to a lower coefficient of thermal expansion—CTE—of the material) was identified on the cooling curves at high temperatures, turning into a section with a steep gradient (higher CTE) at low temperatures. Moreover, a similar cooling curve was obtained for unfilled polyethylene. In the case of the PE composites with ovalene and HBC at N_PAH_ = 44 and 99, the cooling curves have three sections with different gradients, with the maximum gradient observed in the temperature range 320–370 K, where the crystallization process of polyethylene is likely to be occurring. However, no abrupt changes in ρ(T) dependences of PE-based composites were found corresponding to pure crystallization of the polymer matrix.

In general terms, determining the crystallization temperature of a polymer based on computer simulation of the cooling process is an extremely time-consuming task, since polymer crystallization proceeds extremely slowly and requires long simulation times and low cooling rates to study, whereas the cooling rates available for computer simulation of polymer systems are several orders of magnitude higher than the rates used in experimental conditions [77,92].

**Figure 7:**
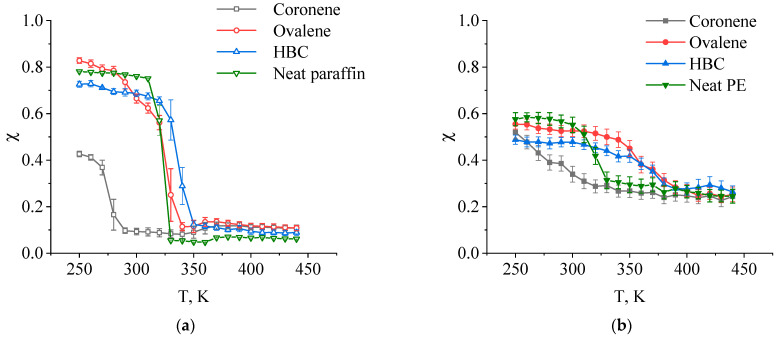
Temperature dependence of the degree of crystallization χ of paraffin (**a**) and PE (**b**) in composites where N_PAH_ = 99.

In order to carry out a more detailed analysis of the processes occurring in the matrix of the composites being studied during cooling, the temperature dependences for the crystallization degree of PE and paraffin for the composites with N_PAH_ = 99 were obtained (Figure 7). For paraffin, a jump in the degree of crystallinity is clearly visible at temperatures in the range of 310–330 K, which corresponds to its crystallization temperature. Moreover, the transition is from systems that are completely disordered to a state with a high degree of crystallinity. The introduction of ovalene and HBC into the composites leads to a slight increase in the crystallization temperature of the paraffin (by 10–20 K). The introduction of coronene, however, has a powerful plasticizing effect on the composites: here there is a sharp decrease in the crystallization temperature of 50 K (up to 270–280 K), with a simultaneous decrease in the maximum degree of crystallinity of the paraffin in these composites.

In the cases of both the unfilled and filled PE, the difference in the transitions observed is more marked. For the unfilled PE there is also a fairly sharp increase in the degree of crystallinity in the temperature range of 310–330 K, and the addition of fillers leads to significant changes to the temperature and crystallization pattern of the polyethylene. The introduction of ovalene and HBC into the composites leads to the same increase in the crystallization temperature of the PE by approximately 40 K: the maximum rate of increase in the degree of crystallinity in these composites is observed at temperatures in the range of 360–370 K. At the same time, the maximum degree of crystallinity is slightly higher for the composites with ovalene than with HBC, which is probably a result of the lower degree of aggregation of ovalene and, as a consequence, fewer steric hindrances in the crystallization of the PE in these systems.

It should be noted that the crystallization temperature of both the PE and the composites based upon it obtained from the results of the computer simulation turns out to be significantly lower than the experimental values determined, for example, using differential scanning calorimetry (DSC), which lie in the range of 390–400 K [43,53]. However, even in the classic DSC version, a strong dependence is observed of the crystallization temperature of the PE upon the cooling rate: an increase in the cooling rate leads to a decrease in the crystallization temperature. Since the cooling rates in the computer simulation are several orders of magnitude higher than the experiment-based rates, the polymer crystallization temperatures in the simulation also turn out to be significantly lower than the temperatures observed in experimental conditions. This conclusion, along with the correctness of the computer simulation results, is confirmed by data obtained using ultrafast scanning calorimetry (UFSC). At cooling rates of the order of 5000 K/s and 10,000 K/s, the peak corresponding to PE crystallization shifts to temperatures of the order of 300 K [42], which is consistent with our results. At the same time, excessively high cooling rates of the systems studied using computer simulation make it impossible neither to detect the crystallization process in the polyethylene itself, nor to correctly determine the temperatures of phase transitions in the system. In the study conducted by M. Mosoabisane et al., at a cooling rate of 2.5·10^10^ K/s, which is 250 times higher than the cooling rate considered in our study, only the glass transition temperature of the polymer was determined, without any signs of a phase transition in the range of the crystallization temperature of the PE [53]. Similarly, our previous work using the example of paraffin crystallization demonstrated the critical importance of choosing the cooling rate for the correct determination of the phase transitions and thermophysical properties of the systems being studied [77].

For the PE filled with coronene, as is the case for similar paraffin-based composites, there is a decrease in the crystallization temperature of the polymer matrix by about 70 K compared with the unfilled sample. However, for these composites, there is on the whole a less dramatic increase in the degree of crystallinity as the temperature decreases, which is also observed in a fairly wide temperature range up to a minimum temperature of 250 K. This behavior can also be thought of as the plasticization of the composite upon the introduction of coronene.

Being the smallest among the considered PAHs, coronene molecules show the least tendency to form aggregates in composites. This fact and the relatively high mobility of coronene molecules resulted in the uniform distribution of this filler in the composites considered. Consequently, both paraffin and PE chains are affected by rather strong steric hindrances, limiting the crystallization process of the matrix in coronene-filled composites.

## 4. Conclusions

A study of paraffin- and polyethylene (PE)-based composites filled with PAH molecules of varying sizes—considered as potential alternatives to graphene or asphaltenes—was conducted using fully atomistic molecular dynamics simulations. These composites allowed us to identify specific features of the aggregation behavior of different carbon fillers, as well as the phase behavior of the composite matrix, depending on the size and concentration of the filler particles.

Similar to blends of paraffin with modified asphaltenes, we found that increasing the size of the polyaromatic core leads to stronger aggregation of the filler particles. In systems containing HBC or high concentrations of ovalene, this aggregation resulted in the formation of a single, columnar aggregate. The aggregation behavior of PAHs was generally similar in both PE- and paraffin-based systems, with one notable exception: in paraffin filled with ovalene, the ovalene molecules embedded into the paraffin crystal structure due to their comparable molecular size. This embedding promoted both increased aggregation of ovalene and a higher degree of paraffin crystallinity compared to other systems. One can anticipate that a similar effect will be exerted by other flat particles possessing in-plane size anisotropy if the geometric dimensions of such particles match the crystallite thickness in the material.

The effect of PAHs on matrix crystallization was similar in PE and paraffin and thus has a general nature. Large-molecule PAHs (ovalene and HBC) increased the crystallization temperature of both PE and paraffin, whereas the small-molecule PAH coronene, which formed numerous small aggregates, significantly decreased the crystallization temperature. However, the fillers’ impact on the degree of crystallinity varied. In PE-based systems at 250 K, the crystallinity showed only a weak dependence on the type and concentration of PAH and was slightly lower than that of unfilled PE. In paraffin-based systems, coronene caused a sharp decrease in crystallinity compared with unfilled paraffin, whereas ovalene slightly increased crystallinity due to the specific structural interactions in these composites. Overall, increasing the filler concentration in paraffin-based composites consistently decreased the matrix crystallinity.

These results demonstrate that the microstructure and phase behavior of polyolefin-based composites can be controllably modified by the addition of specific aromatic nanofillers. Furthermore, the use of commercially available nanofillers in this study lays the groundwork for experimental investigations of similar nanocomposites, enabling direct comparison between computer simulations and experimental data.

## Figures and Tables

**Table 1:** Quantity of paraffin molecules N_par_, PE molecules N_PE_, PAH molecules N_PAH_, mass fraction of PAH ω_PAH_, overall number of atoms N_atoms_ and cubic simulation cell size d_box_ after equilibration at 450 K for the composite systems studied.

Composite	N_par_	N_PE_	N_PAH_	ω_PAH_, %	N_atoms_	d_box_, nm (T = 450 K)
Paraffin + coronene	500	-	44	8.6	32,584	7.25
			99	17.3	34,564	7.44
			214	31.2	38,704	7.57
Paraffin + ovalene	500	-	44	11.0	33,024	7.20
			99	21.8	35,554	7.42
			214	37.7	40,844	7.78
Paraffin + HBC	500	-	44	14.0	33,640	7.21
			99	26.8	36,940	7.49
			214	44.2	43,840	7.91
PE + coronene	-	20	44	8.6	31,624	6.91
			99	17.5	33,604	7.07
			214	31.4	37,744	7.38
PE + ovalene	-	20	44	11.1	32,064	6.94
			99	21.9	34,594	7.13
			214	37.8	39,884	7.98
PE + HBC	-	20	44	14.0	32,680	6.97
			99	27.0	35,980	7.18
			214	44.3	42,880	7.61

## Data Availability

The original contributions presented in this study are included in the article/Supplementary Material. Further inquiries can be directed to the corresponding author(s).

## Supplementary Materials

The following supporting information can be downloaded at: https://www.mdpi.com/article/10.3390/ijms262311509/s1.
