# Supplementary material for: The Impact of Polycyclic Aromatic Hydrocarbons on the Structure and Crystallization Behavior of Nanocomposites Based on Paraffin and Polyethylene
Source: Int J Mol Sci. 2025 Nov 27;26(23):11509. doi: 10.3390/ijms262311509 (PMC12691859; doi:10.3390/ijms262311509)
Supplement: Supplementary file 1 [file ijms-26-11509-s001.zip › ijms-3988148-supplementary.pdf]

Supporting Information

The impact of polycyclic aromatic hydrocarbons on the structure and crystallization behavior of nanocomposites based on paraffin and polyethylene

Sergey V. Larin [1,2,*], Sofia D. Melnikova [1,2], Andrey A. Gurtovenko [1,2], Sergey V. Lyulin [1,2]

1 Branch of the Petersburg Nuclear Physics Institute named by B.P. Konstantinov of the National Research Center "Kurchatov Institute" - Institute of Macromolecular Compounds, Bolshoi Prospect V.O. 31, St. Petersburg 199004, Russia

2 Institute of Chemistry, St. Petersburg State University, 7/9 Universitetskaya Nab., St. Petersburg 199034, Russia

* Correspondence: selarin@macro.ru

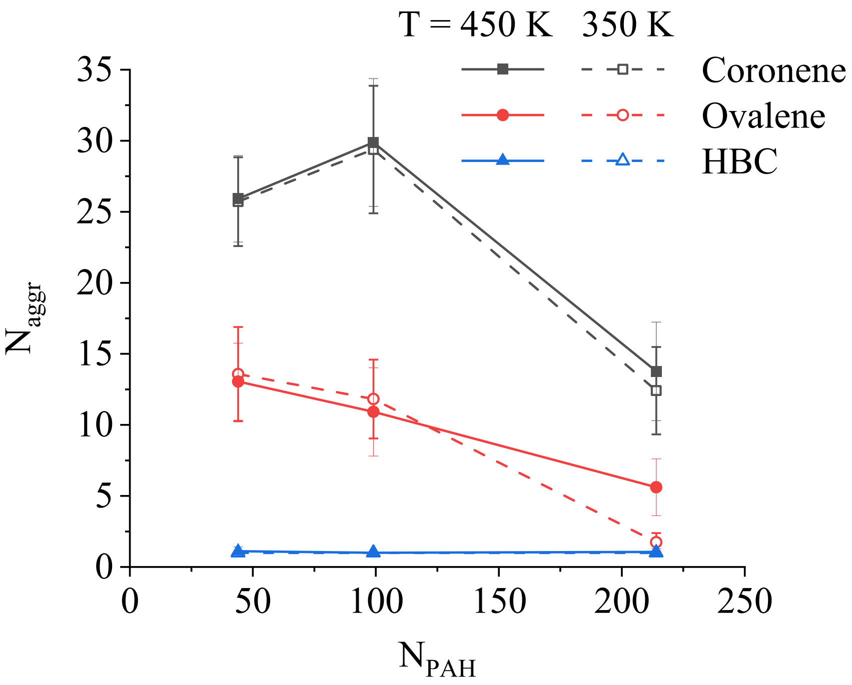

Figure S1. Number of aggregates of coronene, ovalene and HBC in the paraffin at temperatures T = 450 K and T = 350 K.
